# NAFLD: Is There Anything New under the Sun?

**DOI:** 10.3390/ijms18091955

**Published:** 2017-09-12

**Authors:** Amedeo Lonardo, Giovanni Targher

**Affiliations:** 1 Division of Internal Medicine, Department of Biomedical, Metabolic and Neural Sciences, Azienda Ospedaliero-Universitaria, Ospedale Civile di Baggiovara, 41125 Modena, Italy; 2 Section of Endocrinology, Diabetes and Metabolism, Department of Medicine, University and Azienda Ospedaliera Universitaria Integrata of Verona, 37126 Verona, Italy

## 1. Introduction

Nonalcoholic fatty liver disease (NAFLD) is an “umbrella” definition that encompasses a spectrum of histological liver changes ranging from simple steatosis to nonalcoholic steatohepatitis (NASH) with/without fibrosis, “cryptogenic” cirrhosis, and hepatocellular carcinoma (HCC), occurring in a dysmetabolic *milieu*, though in the absence of excessive alcohol consumption and other competing etiologies of chronic liver disease [1].

NAFLD has become a leading cause of end-stage liver disease necessitating liver transplantation and a major cause of HCC in many regions of the world [2]. However, owing to its systemic nature, NAFLD is also strongly associated with the metabolic syndrome [3,4] and excess cardiovascular risk [5]. Over the last 20 years, the amount of scientific information on NAFLD has surged [6], owing to the fact that NAFLD, being closely linked to the so-called “*diabesity*” epidemic [1], has become a major public health problem imposing a substantial clinical and economic burden on many societies worldwide [7,8].

On this background, we felt honored, and happily accepted, to serve as Guest Editors for a monographic special issue of the *IJMS* journal entitled “*Non-Alcoholic Fatty Liver Disease Research 2016*”. Facts have shown that we did the right thing! It was indeed a demanding job, which saw us committed for more than 1 year. Finally, however, this monographic special issue is a true gratification for us and for all those prestigious authors whose thirty-five contributions are published here. We are proud of the participation of so many highly qualified research groups and thus would like to thank each and every one for the time and commitment they dedicated to this outstanding editorial initiative. Similarly, we are also indebted to all reviewers: if not for their expert opinions, this special issue would never have come to light.

## 2. Epidemiology

Although NAFLD has reached pandemic proportions, understanding that there are certain physiological and metabolic factors that can modulate the development and progression of this liver disease may assist physicians in conducting a guided NAFLD screening among high-risk groups of individuals [9,10].

Confirming this paradigm, Losekann et al. examined the prevalence of NASH and risk factors for hepatic fibrosis in 250 patients with morbid obesity submitted to bariatric surgery at a referral center in Southern Brazil [11]. The authors found that hepatic steatosis and NASH were present in nearly 90% and 70% of cases, respectively. Hepatic fibrosis, which affected nearly 45% of these patients, was significantly associated with older age and increased serum alanine aminotransferase and triglyceride levels, thus identifying a subset of morbidly obese patients with more severe liver disease. Finally, the diagnosis of cirrhosis was established in as many as 2% of cases [11]. Collectively, these data further support the notion that physicians should maintain a high index of suspicion that certain high-risk patient groups, such as those with morbid obesity, are more likely to develop fibrosing NASH.

## 3. Diagnosis

By definition, NAFLD still remains a histological diagnosis that requires not only the demonstration of a steatogenic liver disease but also the exclusion of alternative etiologies of chronic liver disease, except for cardiometabolic ones [1,10]. However, given that liver biopsy is an invasive procedure, is costly, and is not completely free of potential risks and acute complications, it cannot be proposed to each individual patient in clinical practice. Moreover, various histological scoring systems are now available. On these grounds, research has addressed multiple non-invasive biomarkers as well as “pros” and “cons” of various histological scoring systems.

Lombardi et al. assessed whether, among the routinely available serum biomarkers, elevated levels of serum uric acid and ferritin may play an additional role as predictors of NAFLD severity [12]. However, based on their revision of the literature, the authors concluded that the power of these two serum biomarkers appears to be too low if considered alone, suggesting that they should best be included in a wider perspective together with other biochemical and metabolic biomarkers in order to predict liver damage noninvasively [12].

Bringing this topic further, Baratta et al. evaluated the role, if any, of the lysosomal acid lipase (LAL) deficiency in diagnosing advanced NAFLD [13]. LAL is a key enzyme responsible for hydrolyzing the cholesteryl esters and triglycerides. In children, Wolman disease is the early onset phenotype of LAL deficiency which rapidly leads to death. Conversely, cholesterol ester storage disease (CESD) is a late onset phenotype that occurs with hepatic steatosis, hepatomegaly, elevated serum aminotransferase levels, and high low-density lipoprotein (LDL)-cholesterol, high triglycerides and low high-density lipoprotein (HDL)-cholesterol levels. Natural history and clinical manifestations of the LAL deficiency in adults are not well defined, and the diagnosis of this disease is often incidental. Based on their review of the literature, Baratta et al. suggested a significant association between reduced LAL activity levels and the pathogenesis and progression of NAFLD [13]. They also pointed out the clinical circumstances under which reduced LAL activity levels should be suspected. 

Consistent with these findings, Shteyer et al., by studying LAL activity levels in patients with microvesicular, idiopathic cirrhosis or NAFLD found that a LAL activity level of 0.5 was the most sensitive for detecting both histologic and non-invasive markers for liver disease severity in these patients [14]. However, additional research is required to better elucidate whether LAL deficiency is a cause or a consequence of advanced hepatic fibrosis, and whether LAL deficiency may be useful for the diagnosis of fibrosing NAFLD.

Despite remarkable advances in non-invasive algorithms developed from tests based on biochemical variables, imaging techniques, or liver stiffness evaluation, the diagnostic phenotype of NAFLD and NASH continues to rely on liver tissue evaluation. NAFLD and NASH are two complex entities, not only for clinical and basic scientists, but also for liver pathologists. Even though much progress has been made, scoring methods gauge injury, but do not replace diagnostic assessment and thus pathologists still need to be trained to identify diagnostic patterns of disease first, and then to apply appropriate scoring systems. Dr. Brunt is among the most experienced liver pathologists worldwide, given that her name is linked to the original proposal for grading and staging NAFLD, which is largely utilized in clinical practice [15] as well as to the Clinical Research Network (CRN) scoring system, which is more appropriate in the research setting [16]. It was, therefore, a pleasure to read her comprehensive review addressing the “pros” and “cons” of the four existent histological scoring systems of NAFLD (i.e., the Brunt proposal for grading and staging; the Clinical Research Network-NASH scoring system; the Fatty Liver Inhibition of Progression (FLIP) algorithm, and the Pediatric NAFLD histologic score), which have specific fields of applications [17]. We fully agree with Dr. Brunt’s conclusions that, as we learn to better use these different histological scoring systems, there remain the expectations for more “pros” and fewer “cons” [17].

## 4. Genetics, Epigenetics, Pathophysiology, and Molecular Pathogenesis

Genetics plays an ever increasingly appreciated pathogenic role in NAFLD development and progression, and an improved understanding of the molecular pathophysiology of NAFLD promises to disclose novel molecular pathways to be manipulated through innovative intervention schemes [1,10]. This is the reason why a substantial proportion of the contributions included in this monographic special issue are devoted to this specific topic.

Telomeres (i.e., repeat DNA sequences located at the terminal portion of chromosomes) shorten during mitosis, thus protecting the tips of chromosomes. Chronic degenerative conditions associated with high cell replication rate are associated with progressive telomere attrition which promotes DNA destabilization and cell aging in mammals, but also disturbed nutrient sensing, which could lead to the development of metabolic disorders such as NAFLD, cryptogenic cirrhosis, and type 2 diabetes mellitus [18,19,20]. In an article by Donati et al., after extensively reviewing the literature the authors concluded that modulation of telomerase or sheltering can be exploited to prevent NAFLD progression, and to define specific treatments for different stages of liver disease [20].

In an article by Ban et al., the authors addressed extracellular vesicles (EVs) as a promising tool for the non-invasive diagnosis of NAFLD [21]. The EVs are submicron membrane-bound structures that play a key role in the cell-to-cell cross talks and are either secreted from stressed and activated cells or formed during apoptosis. Based on the data published in the literature, the authors concluded that it can be reasonably assumed that once EVs become a routinely measured parameter for the assessment of NAFLD, their utility might be further projected to the treatment of the liver disease in its early stages and, potentially, the reversal of NASH [21].

Nuño-Lámbarri et al. reviewed experimental pathology and human NAFLD [22]. These authors further highlighted the importance of both oxidative/nitrative protein stress and mitochondrial dysfunction, which play a major role in stimulating NAFLD damage, as well as the importance of novel non-invasive biomarkers, such as retinol-binding protein-4, lumican, transgelin-2, and hemoglobin, which also play a role in NAFLD pathogenesis [22].

Similar to the oxidative stress, lipidomic analysis also has double significance as a pathogenic and diagnostic. For example, Gambino et al. reported that the elevated levels of serum free fatty acids observed in patients with NAFLD were mainly due to the levels of palmitic and oleic acids (which are the most abundant serum free fatty acids) as well as of those of serum linoleic acid and an imbalance in the n-3/n-6 fatty acids ratio [23].

The gut-liver axis plays a major role in NASH pathogenesis [1]. Consistent with this view, Houghton et al. identified gut microbiota as an emerging key element of personalized medicine and nutrition, and extensively reviewed how lifestyle interventions (diet and physical exercise) may affect gut microbiota, thus influencing the prognosis of NAFLD [24]. Similarly, Machado and Cortez-Pinto also addressed those lines of evidence linking NAFLD with intestinal dysbiosis [25]. They discussed that intestinal dysbiosis may promote the development of obesity through modulation of the energy harvested from the diet, as well as through direct modulation of adipose tissue and hepatic metabolism. Moreover, intestinal bacterial products and perturbed metabolism of choline and bile acids may be hepatotoxic. Dysbiosis can weaken the intestinal barrier, thus allowing bacterial products to invade the bloodstream and inducing systemic chronic inflammation and liver injury [25]. This impressive amount of emerging data on gut microbiota fuels expectations, however, it conflicts with the inadequacy of available studies which are small, heterogeneous, short-term, and do not properly address hepatic histology/risk for progressive liver disease. Hence, the lack of solid evidence still precludes us implementing probiotics in the management of NAFLD or NASH. Extensive preclinical studies comparing different approaches in different animal models of NASH would be important, and fecal microbiota transplantation also deserves further evaluation.

In an article by Aragonès et al., the authors analyzed the hepatic expression of patatin-like phospholipase domain containing 3 (PNPLA3) and other lipid metabolism-related genes in 55 morbidly obese patients (undergoing bariatric surgery) with normal liver histology (*n* = 18), simple steatosis (*n* = 20), and NASH (*n* = 17). These authors found that, compared to patients with normal liver histology, liver PNPLA3 expression was significantly increased in patients with NAFLD. Notably, the hepatic expression of PNPLA3 was even greater in those with NASH. In addition, the expression of the transcription factors liver X receptor (LXR)-α, peroxisome proliferator-activated receptor (PPAR)-α, and sterol regulatory element binding transcription factor (SREBP)-2 was also significantly associated with liver PNPLA3 expression. These findings are compatible with the notion that PNPLA3 is closely related to hepatic fat accumulation, and plays a role in the development and progression of NAFLD [26].

Petäjä and Yki-Järvinen reviewed the pertinent literature aimed at defining how much liver fat content is normal depending on the methods available and at evaluating the cardiometabolic effects of liver fat content as a function of different types of NAFLD (i.e., metabolic-related vs. genetic-related NAFLD) [27]. Based on liver histology, normal liver fat content is defined as macroscopic steatosis in less than 5% of hepatocytes. Based on proton magnetic resonance spectroscopy, normal liver fat content has been defined as ≤5.6%, which corresponds to histologic liver fat of nearly 15%. Whether or not these “normal values” of liver fat content are of clinical relevance with respect to the future development of hepatic fibrosis remains uncertain. NAFLD is a heterogeneous disease. While metabolic-related NAFLD is closely associated with metabolic syndrome features and an increased risk of incident type 2 diabetes and cardiovascular disease, NAFLD caused by either the PNPLA3 or the trans-membrane 6 superfamily member 2 (TM6SF2) genetic variants is usually not accompanied by increased insulin resistance [27]. In other words, it appears that “*not all NAFLD forms were created equal*” in terms of associated cardiometabolic risk [28]. Specifically, addressing the pathogenesis of cardiovascular disease associated with NAFLD, Pisano et al. reported that elevated ferritin levels and mild increased iron stores (i.e., a common finding in patients with NAFLD) may contribute to the development of vascular damage. Moreover, iron depletion may protect from accelerated atherogenesis in both experimental models and human studies [29].

In an article by Machado and Diehl, the authors reported on the Hedgehog (Hh) signaling pathway, which is a known orchestrator of integrated regenerative response by the different cellular players involved in wound-healing. The Hh pathway, which is usually quiescent in the normal liver, will become activated during liver injury. Both experimental and clinical data have consistently confirmed that activation of the Hh pathway mirrors the severity of NASH. Consistently, direct inhibition of the Hh pathway via pharmacological route may prevent liver disease progression in rodent NASH models and, in humans, the Hh pathway activity decreases as NASH improves, thus supporting a promising role of the Hh pathway as a therapeutic target in NASH [30].

Caligiuri et al. reported on the complex and multifactorial nature of NASH pathogenesis, which involves genetic and epigenetic factors; dietary factors; mitochondrial dysfunction and apoptosis; necroptosis; endoplasmic reticulum stress; hypoxia; inflammation; Hh pathway; nuclear receptors; pattern recognition receptors and inflammasomes; adipokines; and gut microbiome [31]. The authors concluded that continuing research is key in providing new targets and biomarkers for the management of NAFLD [31].

In an article by France et al., the authors examined the relationship between liver fat content and indices of lipolysis, and determined whether the degree of lipolysis may reflect insulin resistance or metabolic liver disease [32]. The authors found that glycerol was inversely related to liver fat content, suggesting down-regulation of fatty acid trafficking consistent with the classical paradigm proposed for NAFLD pathogenesis. Levels of ceruloplasmin were also inversely related to liver fat content, which remains an unexplained finding [32].

## 5. Clinical Features and Comorbidities

**Figure 1:**
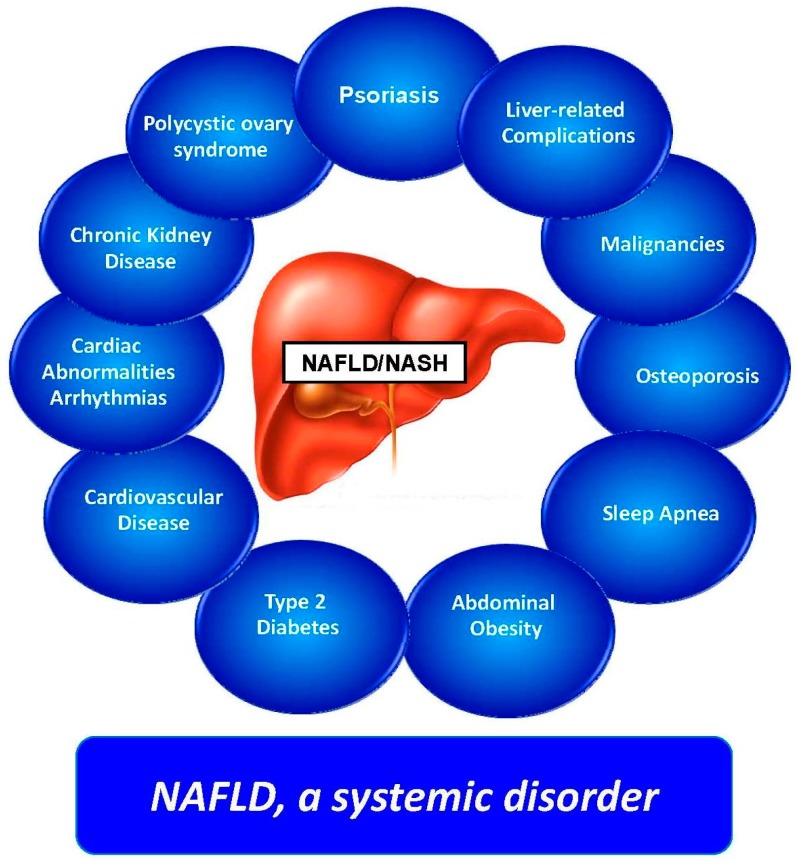
Nonalcoholic fatty liver disease (NAFLD) as a systemic disorder. This figure depicts the ever enlarging protean clinical spectrum of NAFLD. The variety and heterogeneity of the organ systems involved in patients with NAFLD witnesses the systemic nature of this common liver disease. (Modified from [36]).

When addressing clinical features and comorbidities, it must be re-emphasized that NAFLD is a systemic disease [33,34,35] (Figure 1), which is strongly associated with an increased risk of incident fatal and nonfatal cardiovascular events [5,36], and chronic kidney disease [36,37].

The concurrence of chronic plaque psoriasis was almost anecdotally reported in three NAFLD cases observed in 2001 [38], and has now turned into a solid line of research. On this background, it can be better appreciated, as reported by Mantovani et al., that there is now substantial evidence supporting a strong association between the presence and severity of NAFLD and chronic plaque psoriasis, which argues for more careful evaluation and surveillance of NAFLD among patients with psoriasis [39].

The initial paradigm of NAFLD being the “hepatic manifestation of the metabolic syndrome” has undergone a significant evolution and now the relationship between NAFLD and the metabolic syndrome is deemed to be mutual and bidirectional [3,4,36,40]. Wainwright and Byrne highlighted that NAFLD predisposes to the development of metabolic syndrome features, which can, in their turn, increase the risk of development and progression of NAFLD [41]. The authors went further in discussing recent insights from studies of PNPLA3 and trans-membrane 6 superfamily member 2 (TM6SF2) genotypes, which may further contribute to understanding how and why metabolic syndrome features and liver disease are linked in NAFLD [41].

**Figure 2:**
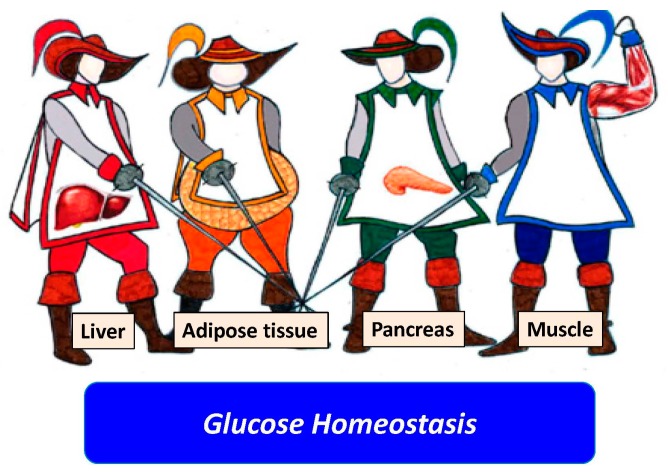
Liver as the fourth “musketeer”. This figure identifies adipose tissue, skeletal muscles, and pancreas as the three key organ systems controlling glucose homeostasis in humans. Together with these three organ systems, the liver also plays a key role in glucose disposal in health. Consistently, a large number of studies based on both the NAFLD and the hepatitis C virus (HCV)-related liver disease spectrum have highlighted the pathogenic role of the liver in the development of type 2 diabetes mellitus. (Modified from ref. [42]).

In their comparative NAFLD-hepatitis C virus review of the literature, Ballestri et al. depicted the liver as the “fourth musketeer” (Figure 2) involved in the pathogenesis of hepatic insulin resistance and type 2 diabetes mellitus [42].

Further attesting to the strong relationship between NAFLD and metabolic syndrome features, Perticone et al., by studying endothelium-dependent vasodilation in nearly 300 never-treated hypertensive patients, suggested that NAFLD is an early marker of endothelial dysfunction in these patients [43].

Clearly, if NAFLD is closely linked with both circulatory endothelial dysfunction and metabolic syndrome features, one might anticipate that NAFLD may also predispose to the development of chronic kidney disease (CKD). In their review of published and ongoing studies, Marcuccilli and Chonchol addressed this interesting topic and concluded that there is now substantial evidence linking NAFLD to CKD development [44]. The mechanisms underlying these two diseases are complexly inter-woven; thus, additional experimental and clinical research is required, including data on both liver and kidney histology. Of interest, lifestyle changes aimed at weight loss and increased physical activity may prevent and benefit both diseases. Finally, physicians’ awareness may lead to screening of CKD among patients with NAFLD and thus to earlier detection and treatment and to improved outcomes in patients with NAFLD and spared organ transplantations [44].

In an article by Villela-Nogueira et al., the authors reviewed the published data on the association between NAFLD and increased aortic stiffness, i.e., a marker of increased cardiovascular risk [45]. Although the underlying biological mechanisms linking NAFLD and increased arterial stiffness remain largely unknown, they possibly involve shared pathways of chronic inflammation and imbalance in adipokine profile [45].

Sanna et al. critically appraised key studies on NAFLD-associated extra-hepatic cancers and speculated on how NAFLD may influence carcinogenesis at these sites [46]. Beyond the increased risk of incident HCC, probably mediated by NASH, substantial epidemiological evidence is now accumulating for a role of NAFLD as a possible risk factor for certain extra-hepatic cancers, particularly in the gastrointestinal tract [46]. Based on the wealth of published data, health care providers taking care of patients with NAFLD should be vigilant for any signs and symptoms suggestive of cancer, particularly colorectal cancers, and promptly refer these patients for further assessment and management whenever indicated.

## 6. Clinical Course and Natural History

The clinical course of NAFLD is characterized by the development of cardiovascular disease and other metabolic comorbidities and by the possible progression of liver disease itself [1,47,48].

Calzadilla Bertot and Adams further highlighted that, although only a small proportion of individuals with NAFLD will develop cirrhosis, the large proportion of the population affected by NAFLD has led to predictions that NAFLD will become a leading cause of end-stage liver disease, liver transplantation, and HCC over the next decade [49]. HCC may arise in non-cirrhotic livers in the setting of NAFLD and is closely associated with the presence of metabolic syndrome and male sex. Along with metabolic syndrome features, other genetic and environmental factors also play a role in the progression of NAFLD [49].

On this background, Gitto and Villa addressed a specific and often overlooked aspect. These authors reported that following liver transplant both recurrent and de novo NAFLD can be found, which usually follows an indolent course with very few cases of liver fibrosis progression [50]. Clinicians should therefore use the diagnosis of NAFLD in the post-liver transplant phase as a marker of increased cardiovascular and CKD risks [50].

## 7. Pediatric Nonalcoholic Fatty Liver Disease 

Compared to the disease as seen in adults, pediatric NAFLD has both similarities and differences [51]. Temple et al. reported that NAFLD affects up to 20% of the general pediatric age-group population, and it is projected to become the major cause of liver pathology, liver failure, and liver transplantation in childhood and adolescence in Western countries over the next decade [52]. However, pediatric NAFLD remains an under-studied, under-recognized, and thus potentially under-managed condition [52].

In their original study Pacifico et al. investigated whether overweight or obese children with NAFLD suffered from impaired renal function, as determined by both estimated glomerular filtration rate and urinary albumin excretion [53]. Data have indeed confirmed that children with NAFLD were at risk for early renal dysfunction. Recognition of this risk in these young patients may help in halting the progression of subclinical kidney disease [53].

## 8. NAFLD and Hepatitis C Virus 

Historically, comparative studies of NAFLD vs. hepatitis C virus (HCV)-related liver disease have been key in promoting an improved understanding of the pathogenesis and natural history of both liver diseases [54,55,56,57].

According to Adinolfi et al., data have shown that hepatic steatosis was a feature of chronic HCV infection and a potentially finalistic condition favoring the persistence and replication of HCV [58]. Hepatic steatosis might thus be a useful marker for identifying those HCV patients at higher risk of liver disease progression, development of extra-hepatic diseases, and, possibly, reduced response rate to novel antivirals [58]. Bringing this consolidated comparative analysis further, Shigefuku et al. aimed at elucidating the difference in liver disease progression (measuring various fibrosis markers, liver function, and hepatic tissue blood flow) in 139 patients with NAFLD and 152 patients with chronic HCV [59]. The authors concluded that, compared to those with HCV-related liver disease, patients with NAFLD exhibited significant changes in hepatic blood flow during the earliest stage of hepatic fibrosis, suggesting that patients with NAFLD need to be followed carefully [59].

## 9. Management

It is now universally agreed that lifestyle changes (diet and physical activity) should be offered to all patients with NAFLD and that treatment of all coexisting cardiometabolic risk factors will often require multiple pharmacological interventions [1,10,60]. Regarding the management of NAFLD, this monographic special issue includes two articles of clinical relevance and two experimental studies.

As regards the role of diet in humans, based on their review of published articles, Stachowska et al. pinpointed that the action of nutrients may be affected by some gene polymorphisms [61]. Therefore, individualization of diet for patients with NAFLD and particularly the nutrient-induced insulin output ratio in people sensitive to fat appears to be a useful tool for determining specific nutritional strategies for patients with NAFLD [61]. Hernandez-Rodas et al., in their turn, extensively reviewed the results of interventions in lifestyle, diet, and behavioral therapies and research results in human, animal, and cell models [62].

Finally, this single-topic special issue also includes two experimental studies. In the first one, Walenbergh et al. reported that subcutaneous injection of 2-hydroxypropyl-β-cyclodextrin could be a useful tool to improve intracellular cholesterol levels in the context of the metabolic syndrome in a mouse model featuring hyperlipidemic low-density lipoprotein (LDL)-receptor knockout animals, possibly through modulation of phytosterols and oxysterols [63]. In the second study, Ideta et al. established a novel NAFLD model mouse, using monosodium glutamate and a high-fat diet, and investigated the effect of teneligliptin (i.e., an oral dipeptidyl-peptidase-4 inhibitor) on the risk of NAFLD progression [64]. They reported that this drug significantly attenuated hepatic lipogenesis by activating 5′ adenosine monophosphate (AMP)-activated protein kinase (AMPK) and down-regulating the expression of multiple genes involved in lipogenesis [64]. However, the clinical relevance of both experimental studies [63,64] remains to be further evaluated.

## 10. Conclusions

We believe that the “*Non-Alcoholic Fatty Liver Disease Research 2016*” monographic special issue of the *IJMS* journal further reinforces the notion that the global health burden of NAFLD is not only confined to progressive liver disease, but also embraces major extra-hepatic complications. In particular, the leading causes of mortality among patients with NAFLD are cardiovascular disease, followed by non-liver malignancy and liver disease. Indeed, NAFLD is a multisystem disease, which by disrupting the regulation of multiple metabolic and inflammatory pathways, plays an important role in the development of cardiovascular disease, type 2 diabetes mellitus, and other metabolic disorders.

Collectively, the published papers provide testimony that NAFLD research has now reached an elevated scientific standard and that most patients with NAFLD will benefit from it. For example, based on the published data, close surveillance of most patients with NAFLD and aggressive management in a subset of them is now highly recommendable. However, further research is needed to better understand the genetic modifiers, natural history, molecular pathogenesis of NAFLD, and biological mechanisms by which NAFLD may contribute to the increased cardiometabolic risk. This promises also to disclose novel and effective treatment strategies for this increasingly prevalent disease, which will ever increasingly impact on the burden of global health in the near future.

## Abbreviations

AMPK	AMP-Activated Protein Kinase
CRN	Clinical Research Network
EVs	Extracellular Vesicles
FLIP	Fatty Liver Inhibition of Progression
HCC	Hepatocellular Carcinoma
Hh	Hedgehog
LAL	Lysosomal Acid Lipase
NAFLD	Nonalcoholic Fatty Liver Disease
NASH	Nonalcoholic Steatohepatitis
PNPLA3	Patatin-Like Phospholipase Domain Containing 3
TM6SF2	Trans-Membrane 6 Superfamily Member 2
